# Uncovering Cellular retinoic acid-binding protein 2 as a potential target for rheumatoid arthritis synovial hyperplasia

**DOI:** 10.1038/s41598-018-26027-x

**Published:** 2018-06-07

**Authors:** Nerea Mosquera, Angela Rodriguez-Trillo, Antonio Mera-Varela, Antonio Gonzalez, Carmen Conde

**Affiliations:** 1 Laboratorio de Reumatología Experimental y Observacional, y Servicio de Reumatología, Instituto de Investigacion Sanitaria de Santiago (IDIS), Hospital Clínico Universitario de Santiago de Compostela (CHUS), SERGAS. Travesía da Choupana s/n, Santiago de Compostela, 15706 Spain; 2 Servicio de Reumatología, Instituto de Investigacion Sanitaria de Santiago (IDIS), Hospital Clínico Universitario de Santiago de Compostela (CHUS), SERGAS. Travesía da Choupana s/n, Santiago de Compostela, 15706 Spain

## Abstract

Rheumatoid arthritis (RA) is a systemic autoimmune disease including synovitis and synovial hyperplasia that contribute to joint destruction. Pivotal pathogenic mechanisms in this process are the dysregulated proliferation and apoptosis of fibroblast-like synoviocytes (FLS). Unfortunately, the mechanisms of FLS dysregulation are not completely elucidated. Here, we explored a new hypothesis based in the potent anti-proliferative and pro-apoptotic activity of retinoids in some types of cancer. Specifically, we investigated the role of retinoids and of the retinoic acid binding proteins, CRABP2 and FABP5, on the proliferation and apoptosis of FLS from RA by adding all-trans retinoic acid (ATRA) or silencing CRABP2 and FABP5. We showed an unconventional behaviour of RA FLS, which were relatively insensitive to ATRA. In effect, ATRA increased the resistance to apoptosis despite the high CRABP2/FABP5 ratio of RA FLS; and *CRABP2* suppression sensitized RA FLS to Fas-induced apoptosis. This latter effect was associated with changes in expression of kinases, *ASK1* up-regulation and *ERK* down-regulation, and increased phosphorylation of JNK. In addition, the potentiation of FLS apoptosis by CRABP2 silencing persisted in the presence of pro-inflammatory mediators, TNF e IL1β. Therefore, the results point to CRABP2 as a potential target to decrease synovial hyperplasia in RA.

## Introduction

Rheumatoid arthritis (RA) is an autoimmune disease characterized by chronic inflammation of peripheral joints that leads to progressive destruction of cartilage and bone. Synovitis and synovial hyperplasia are components of the pathogenic process that include an increase in the number of resident synovial cells such as fibroblast-like synoviocytes (FLS)^1–3^. They increase due to their enhanced proliferation and resistance to apoptosis, which are part of a broader dysregulation of FLS functions of not fully elucidated aetiology. Indeed, it has been shown that RA FLS are resistant to apoptosis despite expression of the functional death receptors Fas/CD95, TRAILR-1, TRAILR-2, and TNFR^4–6^. These changes are important because FLS are pivotal contributors to the severity and chronicity of RA through the production of pro-inflammatory factors that perpetuate the inflammatory process, metalloproteinases that degrade the cartilage, and mediators of bone destruction^7–9^. Therefore, FLS dysregulation provides the opportunity of alternative drug targets to the ones addressed by current RA drugs. These drugs target immune cells and inflammatory cytokines. They include biologics and targeted small molecules and have much improved the prognosis of RA. However, a significant number of patients show a modest or poor response that is only partially improved by changing to drugs against other inflammatory mediators^10,11^. This has motivated the exploration of different molecular pathways in the search of alternatives to inflammation with varied success in preclinical studies, but none of the new targets has yet led to drugs for clinical trials. Here, we have focused our interest in the retinoid pathway.

Retinoids are a group of natural or synthetic organic compounds chemically related to vitamin A (all-*trans*-retinol) that are present in the diet mainly as retinyl esters and metabolized in the body to retinol, retinal and retinoic acid, which is the bioactive form modulating several physiological cellular processes, including differentiation, development, proliferation and apoptosis^12,13^. Interestingly, retinoic acid leads to the differentiation, cell cycle arrest and apoptosis of several types of cells. These actions have been exploited for the treatment of several types of cancer such as acute promyelocytic leukemia, neuroblastoma and Kapossi’s sarcoma^14,15^ and of some skin diseases^16^. Retinoids have also been successful in the treatment of animal models of autoimmune diseases such as sclerosis multiple^17^, and systemic lupus erythematosus^18^. In addition, the effect of retinoid agonists and antagonists has been analysed in several experimental studies of arthritis with diverse results. Whereas the retinoid agonists ATRA^19^ and AM80^20^ improved the clinical and histological scores of collagen-induced arthritis (CIA) accompanied by reduction of anti-collagen antibodies in serum; the 13-cis RA pro-drug or partial agonist was ineffective^20^ or increased the severity of CIA^21^, and the BMS-189453 antagonist reduced arthritis in the CIA and the streptococcal cell wall induced arthritis (SCWA) models^22^. These discordant results remain unexplained and led us to focus on the retinoic acid binding proteins cellular RA-binding protein (CRABP2) and fatty acid-binding protein 5 (FABP5), which are known to exert divergent proliferation/survival roles in the retinoic acid pathway^23–27^. Both binding proteins transport retinoic acid from the cytosol to the nucleus, but whereas CRABP2 delivers retinoic acid to retinoic acid receptors (RARs), FABP5 transport it to the peroxisome proliferator-activated receptor β/γ (PPARβ/γ). This differential receptor specificity has many consequences. Studies in cancer cells have shown that delivery of retinoic acid by CRABP2 facilitates RAR transcriptional activity enhancing differentiation and apoptosis, and reducing proliferation^23,24^. On the contrary, transport of retinoic acid by FABP5 promotes survival^25–27^. In accordance with these differential effects, it has been observed that the pro-apoptotic or pro-survival role of retinoic acid correlates with the CRABP2/FABP5 ratio in a variety of cells, making it possible that modulation of this pathway could have impact in arthritis. This hypothesis is supported by the increased expression of the *Crabp2* gene in joint tissues and synovial fluid of arthritic mice observed in previous studies from our laboratory^28^ and from other group^29^. The increase was observed 7 to 18 days after injection in the K/BxN serum transfer model, a time span including the peak of inflammation at 9 days and the phase of resolution, which could indicate a role of Crabp2 in the auto-limitation of arthritis. In the present study, we have found that RA FLS are unconventional cells, relatively insensitive to ATRA and independent of the CRABP2/FABP2 ratio. In addition, ATRA exacerbated FLS apoptosis resistance, making it unsuitable as drug candidate, whereas CRABP2 silencing potentiated apoptosis, making it a potential drug target.

## Results

### Effect of ATRA on proliferation and apoptosis of RA FLS

**Figure 1:**
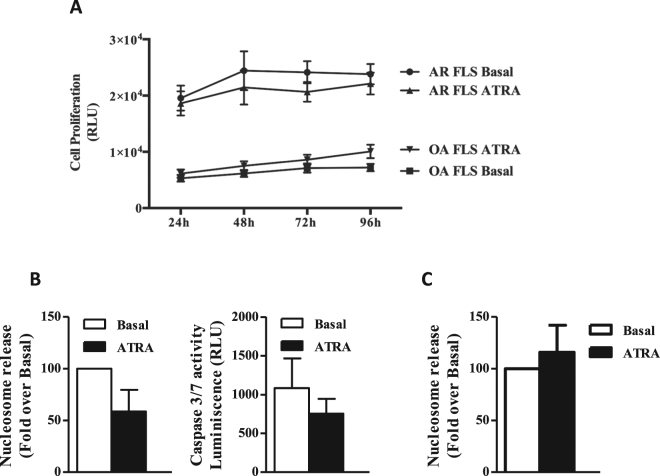
Effect of All-trans retinoic acid (ATRA) on the proliferation of RA FLS. (**A**) Proliferation of RA and OA FLS stimulated with or without 5 μM ATRA. Values are the mean (SEM) of 11 different RA and 5 OA FLS lines. (**B**) Apoptosis of RA FLS following stimulation with 5 μM of ATRA was quantified by nucleosomal release enzyme-linked immunosorbent assay (ELISA) and shown as relative to the Basal cell value. Analysis of caspase 3/7 activity as relative luminescence units (RLU) are shown in right panel. Values are the mean ± SEM of FLS from 6–8 RA patients. (**C**) Apoptosis of OA FLS after stimulation with 5 μM of ATRA quantified by nucleosomal release ELISA. Data are given as percentage of control, considered as 100%. Values are the mean ± SEM of FLS from 5 OA patients.

We have analysed the effect of ATRA on the proliferation of RA FLS for up to 96 h. As shown in the Fig. 1A, we did not find significant changes in the analysis of the proliferation curve in spite of including 11 different RA lines (two-way repeated-measures ANOVA). These results were similar to the found in control OA FLS (Fig. 1A), which showed lower basal proliferation unmodified by exposure to ATRA.

Next, we analysed RA FLS spontaneous apoptosis in the presence of ATRA with two independent methodologies (Fig. 1B). Results of the two assays, nucleosome release and caspases 3/7 activity, were concordant in showing no significant increase in FLS spontaneous apoptosis (Wilcoxon matched-pairs tests). On the contrary, there was a nominal decrease (Fig. 1B). The absence of apoptosis increase was not RA specific as it was also observed in OA FLS (Fig. 1C). Overall, these data seem to indicate the ATRA lack of effect on proliferation and spontaneous apoptosis are FLS inherent.

**Figure 2:**
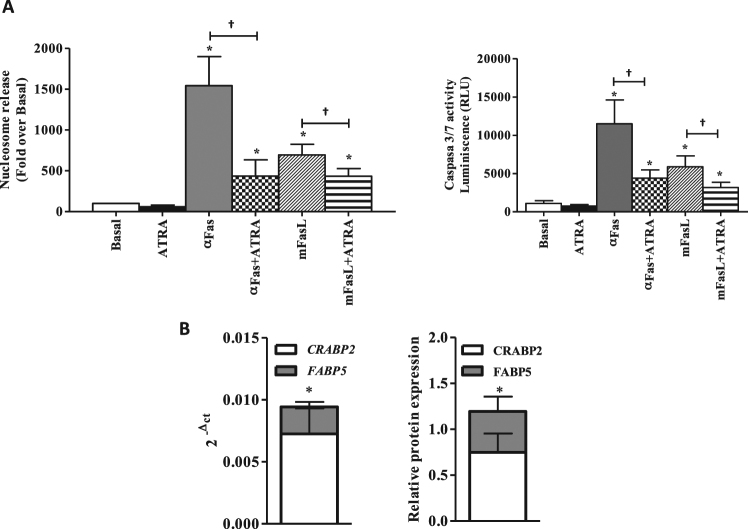
Effect of ATRA on the spontaneous and Fas-induced apoptosis of RA FLS. (**A**) Fas-induced apoptosis of RA FLS following stimulation with 5 μM of ATRA + anti-Fas (0.5 μg/ml) or 5 μM of ATRA + memFasL(100 ng/ml) was quantified by nucleosomal release enzyme-linked immunosorbent assay and shown as relative to the Basal cell value. Analysis of caspase 3/7 activity as relative luminescence units (RLU) are shown in right panel. Values are the mean ± SEM of FLS from 6–8 RA patients. ^†^ indicates p < 0.05 and * indicates p < 0.05 versus basal, by Wilcoxon matched-pairs test. (**B**) CRABP2 and FABP5 protein expression assessed by western blot. A representative blot is shown. Results are the mean (SEM) of 7 different RA FLS lines. * indicates p < 0.05, by Mann-Whitney U test.

To study more thoroughly the effect of ATRA on the apoptosis of RA FLS, we analysed the effect of ATRA on the apoptosis induced by Fas signalling (Fig. 2A). In this experiment, RA FLS were treated with 0,5 μg/ml of anti-Fas antibody or with 100 ng/ml of memFasL for 24 hours in the presence of ATRA. As expected, stimulation of the Fas pathway resulted in increases in FLS apoptosis measured either as nucleosome release or as capases 3/7 activity that were of 26 fold with anti-Fas antibody and of 15 fold with memFasL. Contrary to the observed in other cell types, ATRA did not potentiate the Fas induced apoptosis but was opposed to it. The results showed that apoptosis induced by anti-Fas antibody was reduced to about a third and the induced by memFasL to about half of the levels in the absence of ATRA (Fig. 2A). These results indicate that ATRA acts as a survival factor in RA FLS.

Searching for an explanation to these results, we analysed the mRNA and the protein expression of CRABP2 and FABP5 in RA FLS. As shown in Fig. 2B, both proteins were expressed in FLS from RA patients, with a clearly predominant expression of CRABP2 over FABP5, which excluded the simple participation of this ratio in ATRA associated apoptosis resistance.

### Exacerbated RA FLS sensitivity to apoptosis mediated by Fas by *CRABP2* silencing

**Figure 3:**
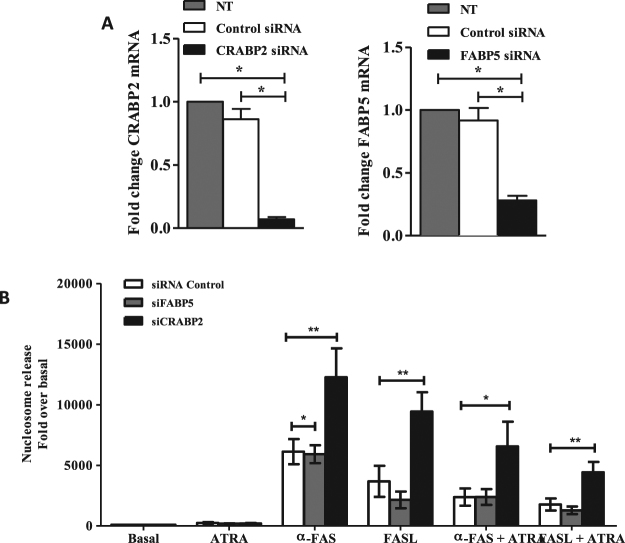
*CRABP2* suppression sensitizes RA FLS to Fas-induced apoptosis. (**A**) RA FLS were transfected with CRABP2, FABP5 or non-silence control siRNA, total RNA were extracted and real-time PCR were performed. (**B**) Apoptosis of RA FLS following pre-treatment with 5 μM of ATRA and stimulation with 0.5 μg/ml of anti-Fas or 100 ng/ml memFasL was quantified by nucleosomal release enzyme-linked immunosorbent assay and shown as relative to the Basal cell value. Values are the mean ± SEM of FLS from 7–9 RA patients. * indicates *p* < 0.05, ** indicates *p* < 0.01, ^#^ and ^‡^ indicates *p* < 0.05 versus anti-Fas-only treatment, and ^†^ indicates *p* < 0.05 versus memFasL-only treatment, by Wilcoxon matched-pairs test.

The lack of concordance of the CRABP2/FABP5 ratio with the results observed in RA FLS could be due to a change in the association of these binding proteins with apoptosis signalling. Thus, we analysed the effect of *CRABP2* and *FABP5* silencing on Fas-induced apoptosis. We transfected FLS from RA patients with specific CRABP2, FABP5 or control siRNA and we checked the silencing of CRABP2 and FABP5 mRNA expression by real-time PCR. CRABP2 siRNA reduced by more than 90% *CRABP2* transcription (Fig. 3A). Similarly FABP5 mRNA expression was reduced by 72% after FABP5 siRNA transfection. Supressed FLS were stimulated with 0.5 μg/ml of anti-Fas antibody or 100 ng/ml of memFasL for 24 hours and apoptosis was determined. As shown in Fig. 3B, a significant increase of nucleosome release was observed in RA FLS lacking *CRABP2* after stimulation with anti-Fas antibody, which was about three fold the observed in FLS transfected with control siRNA (p = 0.0039). Similar results were observed with memFasL (p = 0.0078) (Fig. 3B). These effects were confirmed when apoptosis was assessed as caspase 3/7 activity (data not shown). By contrast, *FABP5* silencing showed a statistical significant decrease of apoptosis in the RA FLS stimulated with anti-Fas antibody (Fig. 3B, p = 0.03), although the reduction was small and not significant after treatment with memFasL (Fig. 3B). The increased apoptosis in absence of CRABP2 persisted in the presence of added ATRA, although, the level of apoptosis was reduced to about half, both in controls and in the silenced cells (Fig. 3B). The small reduction after FABP5 silencing, in this condition of added ATRA, did not persist (Fig. 3B).

### *ASK1* up-regulation and *ERK* down-regulation in RA FLS lacking *CRABP2*

**Figure 4:**
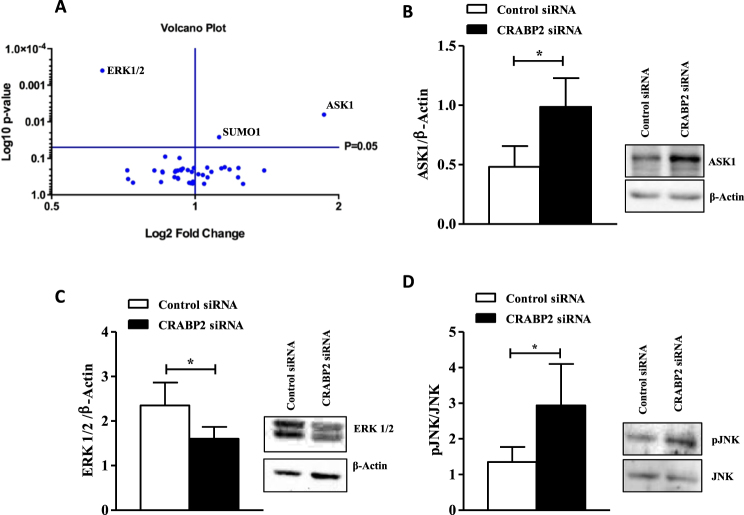
*CRABP2* suppression increases the expression of ASK1 and JNK activity and reduces the expression of ERK1/2. RA FLS were transfected with control or CRABP2 siRNA and stimulated with 0.5 μg/ml of anti-Fas and ASK1 (**A**), ERK1/2 (**B**) and pJNK (**C**) expression were determined by Western blotting (representative blots at right) with densitometric quantification of band intensity. Values are the mean ± SEM from 5–7 RA patients. * indicates *p* < 0.05, by Wilcoxon matched-pairs test. For full western blots please see the supplementary information.

The mechanisms underlying the sensitization to Fas-induced apoptosis of RA FLS lacking *CRABP2* were explored by expression analysis of 45 Fas apoptosis-related genes. FLS from three RA patients were stimulated or not with 0.5 μg/ml of anti-Fas antibody for 12 hours and their extracted mRNA was assessed with a PCR array. Genes showing ≥1.5-fold change and p < 0.05 in the statistical test were considered with differential expression, as shown in Volcano plot (Fig. 4A). Two kinase genes were in this group, apoptosis signal-regulating Kinase 1 (*ASK1*) that was up regulated, and extracellular signal-regulated kinases (*ERK*), that was down regulated (*ASK1*, fold change: 1,86, *p* = 0.0064 and *ERK*, fold change: 0.64, *p* = 0.0004). The remaining 46 genes in the array did not fulfil the two criteria for differential expression. The differential expression of ASK1 and ERK was confirmed at the protein level by western blot analysis, where ASK1 showed a 49% higher expression (Fig. 4B) and ERK1/2 a 27% lower expression (Fig. 4C) in CARBP2 suppressed RA FLS compared with RA FLS transfected with control siRNA. As ASK activates JNK by phosphorylation during apoptosis initiated by death receptors, we have analysed the status of this kinase. The level of phosphorylated JNK was significantly increased after treatment with anti-Fas antibody in the CRABP2 suppressed RA FLS in a way consistent with the activation of the pathway including activation of ASK1 (Fig. 4D, *p* = 0.03).

### Effect of inflammatory mediators in the Fas-induced apoptosis of RA FLS

**Figure 5:**
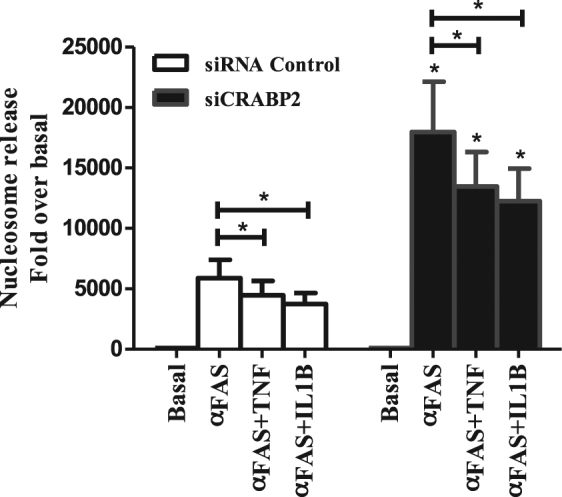
Abrogation of the increased apoptosis in RA FLS lacking *CRABP2* by TNF or IL1β inflammatory mediators. Nucleosome release of RA FLS with siRNA silenced CRABP2 following anti-Fas antibody (αFas) 0.5 μg/ml stimulation after pre-treatment with 10 ng/ml of TNF or 1 ng/ml of IL-1β. Values are the mean ± SEM of cells from 6 RA patients. * indicates *p* < 0.05, by Wilcoxon matched-pairs test.

Given the unconventional behaviour of RA FLS we wonder whether the inflammatory environment could influence the potential beneficial effect of *CRABP2* silencing. To explore this question, we repeated the experiment of stimulation of the Fas pathway with anti-Fas antibody in CRABP2 suppressed RA FLS in the presence of TNF or of IL1β, which are pivotal pro-inflammatory mediators. Apoptosis was determined 24 h after the addition of anti-Fas antibody with the nucleosome release assay. As shown in Fig. 5, incubation in the presence of TNF or of IL1β reduced the cell death induced by Fas by about 25%, both in RA FLS lacking *CRABP2* and in siRNA controls cells. This result suggests the two cytokines promote FLS resistance to apoptosis, but with independence of the function of CRABP2.

## Discussion

Here, we have found that contrarily to expectations, ATRA does not show beneficial effects on proliferation and survival of human RA FLS. In addition, we have observed that this behaviour of RA FLS was not explained by a low CRABP2/FABP5 expression ratio, as in other cell types, but by an idiosyncratic phenotype. This phenotype included exacerbated Fas-induced apoptosis in the absence of CRABP2 as the most remarkable trait. This beneficial effect persisted in the presence of the survival signals of inflammatory mediators, making of CRABP2 a new potential drug target to address FLS hyperplasia in RA.

Our initial hypothesis was that ATRA would help control the dysregulated proliferation and resistance to apoptosis characteristic of RA FLS. This hypothesis was based on the effects demonstrated by ATRA and other retinoids in some skin diseases and in cancer such as acne, psoriasis or acute promyelocytic leukemia^30^. However, progress to wider use has not been possible due to poor results in clinical trials, which are attributed to a variety of causes including the high frequency of cancer cells that are resistant^31,32^. The causes of resistance are very varied and including loss or overexpression of RA receptors^31,33^, inactivation of ATRA, block of the formation of RAR:RXR heterodimers, epigenetic silencing of RARβ1 (in the old bibliography referred as RARβ2); and cytoplasmic sequestration of retinoids by CRABP1 or CRABP2 transport proteins^31,32,34,35^. Another demonstrated mechanism of resistance to anti-proliferation and to apoptotic signals is based in the differential delivery of retinoids to nuclear receptors by CRABP2 or FABP5^26,27^. These two intracellular transport proteins show different abundance ratios in each cell type, and their retinoid cargo is directed either to RAR/RXR or to PPARβ/δ/RXR heterodimers, respectively. Signalling through the latter receptors enhances cell proliferation and survival^26,27^. This CRABP2/FABP5 ratio reached pre-eminence a few years ago as a mechanism to explain response to retinoids, but current evidence supports a complex panorama with many possible mechanisms in interplay and with lack of demonstrable participation of the CRABP2/FABP5 ratio in the retinoic acid response of some cells^30–32,35^. Therefore, the resistance of RA FLS to the anti-proliferative and pro-apoptotic effect of ATRA we have observed is unconventional, but not without abundant precedent. Specifically, increased survival induced by retinoids after Fas pathway stimulation has been clearly demonstrated in T cells^36–38^. Similarly, the absence of correlation of resistance with the CRABP2/FABP5 ratio has also been repeatedly observed^39^.

Further example of the CRABP2/FABP5 role uncovered the most surprising findings of our work. First, FABP5 silencing did not increase apoptosis either at its spontaneous level or stimulated with Fas signalling. These results reinforced the independence of FLS resistance to ATRA form the CRABP2/FABP5 ratio. They could be considered contrary to the model of differential delivery to RAR/PPARβ/δ, but still they could have been explained as the consequence of the predominant expression of CRABP2. This possibility was negated by the observation that CRABP2 silencing did not modify spontaneous apoptosis and augmented markedly the number of cells dying on Fas stimulation. To add complexity the effects on cell survival of silencing either FABP5 or CRABP2 were independent of added ATRA. Therefore, at this level, it become compelling to consider that RA FLS are idiosyncratic in their retinoic acid pathway. However, it is important to consider some limitations and precedents. On the side of the limitations, retinoids plus Fas signalling or retinoid independent effects of the transporters have not been studied very thoroughly making it possible that other yet unexamined cells show a similar phenotype. On the side of the precedents, Donato LJ *et al.*^40^ have described ligand-independent effects of CRABP2 in inducing apoptosis of cells from mammary cancer. The Noy team further has described this second function of CRABP2 as mediated by the RNA-binding and stabilizing protein HuR and completely independent from the presence of retinoic acid^41^. Perhaps of more relevance, two recent reports have demonstrated an anti-apoptotic function of CRABP2^34,42^. In the first, CRABP2 knockdown sensitized glioblastoma cells to retinoic acid induced apoptosis. This effect was attributed partly to cytoplasmic sequestration of retinoic acid by CRABP2 and to induction of the anti-apoptotic CRYAB small heat shock protein^34^. To investigate if this mechanism was involved in RA FLS, we analysed the expression of CRYAB after silencing CRABP2, but no differences were found (data not shown). In the second report, there is precedent for the two unconventional aspects of our results, RA independence and increased apoptosis with CRABP2 down-regulation. In effect, silencing of CRABP2 in the absence of retinoic acid decreased proliferation and induced apoptosis in malignant peripheral nerve sheath tumors cells^42^. In addition, a reduction of viability without detectable apoptosis was observed in a variety of cells including normal and neurofibroma-derived fibroblasts^42^. Mechanisms of the reduced viability or of the increased spontaneous apoptosis were not clarified, although induction of the type 1 interferon pathway was observed. Therefore, the idiosyncratic role of CRABP2 in RA FLS has some precedent in other cell types, with the most resembling observed in other fibroblasts.

We were able to elucidate some of the mechanisms underlying the enhanced sensitivity to Fas-induced apoptosis of RA FLS lacking CRABP2 thanks to a PCR array of genes on the Fas apoptosis pathway. The upregulation of *ASK1* expression and the downregulation of *ERK* are consistent with the potentiation of apoptosis. In effect, Fas signalling activates the Jun NH2-terminal kinase (JNK) pathway through interaction of the receptor-associated protein Daxx with ASK1, which is a MAP kinase kinase kinase that becomes activated and phosphorylates JNK^43^. Evidence of these steps was confirmed by our demonstration of increase JNK phosphorylation. The phosphorylated JNK translocates to the nucleus where it can potentially lead to an increase in the expression of pro-apoptotic genes, as FasL or Bak, and a decrease in the expression of pro-survival genes^43^. However, it is likely that for RA FLS the most relevant effect of activated JNK is activation of the mitochondrial pathway^44,45^ because RA FLS are type II cells in which death-receptor-induced apoptosis requires activation of the mitochondrial branch^46,47^. JNK readily translocates to mitochondria, where it is critical for the release of cytochrome C from the inner membrane space, triggering apoptosis^43^. Thus, up-regulation of ASK1 and resulting JNK activation contributes to the potentiation of apoptosis in RA FLS lacking CRABP2. In a similar way, we interpret the reduced ERK expression as a factor that contributes to the phenotype given that ERK is known to interfere with Fas-induced apoptosis^48,49^.

Potentiation of Fas-induced apoptosis by CRABP2 silencing could be of clinical interest because it persisted in the presence of TNFα and IL1β, two pro-inflammatory stimuli that are present in arthritis and that increase RA FLS survival. The role of TNFα is striking because in many cell types it induces cell death, but it is common observation that RA FLS respond with proliferation and survival^50,51,52^. This response could be related with NFκB activation that provide strong survival signals and is very marked in RA synovial cells. However, other FLS specific mechanisms have been suggested as the induction of soluble Fas^53^. In addition, the potential applicability of CRABP2 to potentiate RA FLS apoptosis is reinforced by the expression of Fas in the synovial lining^5,6^ and the finding that hydroxychloroquine, which is a weak disease-modifying anti-rheumatic drug (DMARD), potentiates Fas induced apoptosis of RA FLS^54^, and because therapeutic potentiation of the Fas pathway has consistently been found beneficial in both animal arthritis models and in human RA FLS^55^.

Overall the data shown here indicate that the retinoid pathway in RA FLS shows a complex and idiosyncratic regulation. As a consequence, ATRA does not show the conventional antiproliferative and pro-apoptotic effects excluding its use as potential drug to address synovial hyperplasia and activation. However, the potentiation of Fas induced apoptosis observed after suppression of CRABP2 that persists in spite of an inflammatory cytokines makes of this molecule a potential drug target worth of study in additional preclinical models of arthritis.

## Material and Methods

### Patients and cell culture

FLS were derived from synovial tissue obtained from 11 patients with RA undergoing synovectomy by clinical indication, always associated with acute synovitis; and 5 patients with OA at the time of total joint replacement. The RA patients fulfilled the American College of Rheumatology (ACR) criteria for the classification of RA^56^ and provided informed written consent. FLS were obtained by digestion of synovial tissue as previously described^46^. Adherent cells at 80–90% confluence were trypsinised and diluted at a 1:3 split ratio. FLS from passages 3 to 8 were used for all experiments.

### Ethics Statement

The study was performed according to the recommendations of the Declaration of Helsinki and was approved by the Comité Ético de Investigación Clínica de Galicia. Approval No. 2014/393.

### Small interfering RNA transfection

RA FLS cells were cultured in six-well plates (14 × 10^4^ cells/well) or in P100 plates (7 × 10^5^ cells/plate) in DMEM medium 10% FBS and 1% glutamine. Cells were transiently transfected with 20 nM of ON-TARGET plus Human siRNA-SMART pools against CRABP2 and against FABP5, or with ON-TARGET plus Non-targeting Pool, as control siRNA, all of them from Dharmacon (GE, UK). Transfections were performed using DharmaFECT 1 (Dharmacon) in Opti-MEM (Life Technologies, Thermo Fisher Scientific, MA, USA). Experiments were performed 72–144 h after siRNA transfection, as indicated. The degree of suppression was determined by real-time quantitative polymerase chain reaction (qPCR) and by Western blotting.

### Proliferation assay

RA FLS were cultured in 96-well plates (2 × 10^3^ cells/well) with DMEM, 5% FBS, 1% glutamine and 1% penicillin/streptomycin. Cells were treated with 10 ng/ml of TNF or 5 μM of all-trans retinoic acid (ATRA) or vehicle (0,05% DMSO in DMEM) for 24, 48, 72 and 96 hours and proliferation was determined using the CellTiter-Glo luminescent viability assay (Promega, Wisconsin, USA) according to the manufacturer’s instructions.

### Apoptosis assays

RA FLS were cultured in 96-well plates (6 × 10^3^ cells/well) with DMEM, 10% FBS, 1% glutamine and 1% penicillin/streptomycin. After 12 h of serum-deprivation cells were treated with anti-Fas antibody (0.5 µg/ml) or memFas ligand (100 ng/ml) or medium for 24 hours in the presence of ATRA 5 μM or its diluent, or in the presence of 10 ng/ml of TNF. Apoptosis was determined by quantifying mono- and oligo-nucleosomal DNA using a cell death detection enzyme-linked immunosorbent assay (ELISA) KIT (Roche Diagnostics, Switzerland) according to the manufacturer’s instructions. Apoptosis was confirmed by analysis of activated caspases 3/7 using a Caspase-Glo 3/7 assay (Promega) following manufacturer’s instructions.

### Real-time qPCR

Total RNA was obtained using the Speedtools total RNA extraction Kit (Biotools, Madrid, Spain) according to the manufacturer’s instructions. Real-time qPCR was performed in duplicate in a Mx3005P real-time qPCR system, using 1-Step QRTPCR-Brilliant III SYBR Green (Agilent Technologies, CA, USA), according to the manufacturer’s protocol. Relative levels of gene expression were normalized to the β-actin gene using the comparative C_t_ method, where C_t_ is the cycle at which the amplification is initially detected. The relative amount of mRNA was calculated according to the 2^−ΔΔCt^ method, where: ΔC_t_ = C_t target_ − C_t β-actin_ and ΔΔC_t_ = (C_t target_ − C_t β-actin_)_NT_ − (C_t target_ − C_t β-actin_)_experimental siRNA._

For the not transfected (NT), ΔΔC_t_ = 0, and 2° = 1. For the experimental siRNA, the value 2^−ΔCt^ indicates gene expression relative to β-actin and the value 2^−ΔΔCt^ indicates the fold change in gene expression relative to the control siRNA. The specific primers to quantify human CRABP2, human FABP5, and human β-actin were from Qiagen (Hilden, Germany).

### Western blot analysis

RA FLS were cultured, transfected and/or treated, as indicated and proteins were extracted using cell lysis buffer. The protein concentration was determined by Bradford assay (Bio-Rad Protein Assay; Bio-Rad, CA, USA). Protein samples (20 µg) were resolved in 8% gradient SDS-PAGE, transferred onto PVDF membranes (Merck Millipore, Darmstadt, Germany) and probed with primary antibodies directed against CRABP2 (generously provided by Cécile Rochette-Egly, IGBMC, Illkirch, France), FABP5 (R&D Systems MN, USA), ASK1 (sc-5294), ERK (sc-93, both from Santa Cruz Biotechnology, CA, USA) and β-actin (AC-74, Sigma-Aldrich, St.Louis, MO, USA). Bound antibodies were revealed with horseradish peroxidase-couple secondary anti-rabbit or anti-mouse antibodies (both from Santa Cruz Biotechnology).

### Fas apoptosis PCR array

The relative expression of 48 Fas apoptosis-related genes was assessed using the Taqman array plate Fas signaling (Life Technologies, Thermo Fisher Scientific). RA FLS (7 × 10^5^ cells/well in P100 plate) were silenced and treated with 0.5 µg/ml of anti-Fas for 12 hours. Total RNA was isolated as detailed above, and the complementary DNAs (cDNAs) were reverse transcribed from 2.7 µg of RNA using the High-Capacity cDNA Reverse Transcription Kit (Life Technologies) according to the manufacturer’s instructions.

### Statistical analyses

Differences between experimental groups were assessed by Two-way repeated-measures ANOVA, Mann-Whitney U or Wilcoxon matched-pairs tests. P values less than 0.05 were considered significant. All analyses were performed with GraphPAD Prism (GraphPAD Software, San Diego, CA).

### Data availability

All data generated during this study are available upon request from the authors.

## Electronic supplementary material

Dataset 1

## References

[1] McInnes IB, Schett G (2011). The pathogenesis of rheumatoid arthritis. N. Engl. J. Med..

[2] Klareskog L, Catrina AI, Paget S (2009). Rheumatoid Arthritis. Lancet.

[3] Firestein GS, McInnes IB (2017). Immunopathogenesis of Rheumatoid Arthritis. Immunity.

[4] Korb A, Pavenstädt H, Pap T (2009). Cell death in rheumatoid arthritis. Apoptosis.

[5] Nakajima T (1995). Apoptosis and functional Fas antigen in rheumatoid arthritis synoviocytes. Arthritis Rheum..

[6] Asahara H (1997). Expression of Fas antigen and Fas ligand in the rheumatoid synovial tissue. J. Rheumatol..

[7] Andersson AK, Li C, Brennan FM (2008). Recent developments in the immunobiology of rheumatoid arthritis. Arthritis Res. Ther..

[8] Noss EH, Brenner MB (2008). The role and therapeutic implications of fibroblast-like synoviocytes in inflammation and cartilage erosion in rheumatoid arthritis. Immunol. Rev..

[9] Huber LC (2006). Synovial fibroblasts: key players in rheumatoid arthritis. Rheumatology (Oxford).

[10] Chen, Y. F. *et al*. A systematic review of the effectiveness of adalimumab, etanercept and infliximab for the treatment of rheumatoid arthritis in adults and an economic evaluation of their cost-effectiveness. *Health Technol. Assess*. **10**(42), iii–iv, xi–xiii, 1–229 (2006). 10.3310/hta10420 17049139

[11] Rubbert-Roth A, Finckh A (2009). Treatment options in patients with rheumatoid arthritis failing initial TNF inhibitor therapy: a critical review. Arthritis Res. Ther..

[12] Al Tanoury Z, Piskunov A, Rochette-Egly C (2013). Vitamin A and retinoid signaling: genomic and nongenomic effects. J. Lipid Res..

[13] Coyle KM, Sultan M, Thomas ML, Vaghar-Kashani A (2013). & Marcato P. Retinoid signaling in cancer and its promise for therapy. J Carcinog. Mutagen..

[14] Altucci L, Gronemeyer H (2001). The promise of retinoids to fight against cancer. Nat. Rev.Cancer.

[15] Soprano DR, Qin P, Soprano KJ (2004). Retinoic acid receptors and cancers. Annu. Rev. Nutr..

[16] Beckenbach L, Baron JM, Merk HF, Löffler H, Amann PM (2015). Retinoid treatment of skin diseases. Eur. J. Dermatol..

[17] Massacesi L (1991). Immunosuppressive activity of 13-cis-retinoic acid and prevention of experimental autoimmune encephalomyelitis in rats. J. Clin. Invest..

[18] Perez de Lema G (2004). Retinoic acid treatment protects MRL/lpr lupus mice from the development of glomerular disease. Kidney Int.

[19] Kwok S-K (2012). Retinoic acid attenuates rheumatoid inflammation in mice. J. Immunol..

[20] Kuwabara K, Shudo K, Hori Y (1996). Novel synthetic retinoic acid inhibits rat collagen arthritis and differentially affects serum immunoglobulin subclass levels. FEBS Letters.

[21] Trentham DE, Brinckerhoff CE (1982). Augmentation of collagen arthritis by synthetic analogues of retinoic acid. J. Immunol..

[22] Beehler B (2003). Inhibition of disease progression by a novel receptor antagonist in animal models of arthritis. J. Rheumatol..

[23] Donato LJ, Noy N (2005). Suppression of mammary carcinoma growth by retinoic acid: proapoptotic genes are targets for retinoic acid receptor and cellular retinoic acid-binding protein II signaling. Cancer Res..

[24] Budhu AS, Noy N (2002). Direct channeling of retinoic acid between cellular retinoic acid-binding protein II and retinoic acid receptor sensitizes mammary carcinoma cells to retinoic acid-induced growth arrest. Mol. Cell. Biol..

[25] Tan NS (2002). Selective cooperation between fatty acid binding proteins and peroxisome proliferator-activated receptors in regulating transcription. Mol. Cell. Biol..

[26] Schug TT, Berry DC, Shaw NS, Travis SN, Noy N (2007). Opposing effects of retinoic acid on cell growth result from alternate activation of two different nuclear receptors. Cell.

[27] Schug TT (2008). Overcoming retinoic acid-resistance of mammary carcinomas by diverting retinoic acid from PPAR beta/delta to RAR. Proc. Natl. Acad. Sci. USA.

[28] García S, Forteza J, López-Otin C, Gómez-Reino JJ, González A (2010). Matrix metalloproteinase-8 deficiency increases joint inflammation and bone erosion in the K/BxN serum-transfer arthritis model. Arthritis Res. Ther..

[29] Jacobs JP (2010). Deficiency of CXCR2, but not other chemokine receptors, attenuates autoantibody-mediated arthritis in a murine model. Arthritis Rheum..

[30] Uray IP, Dimitrovsky E, Brown PH (2016). Retinoids and rexinoids in cancer prevention: from laboratory to clinic. Seminars Oncol..

[31] Garattini E (2014). Retinoids and breast cancer: From basic studies to the clinic and back again. Cancer Treat. Rev..

[32] Coyle, K. M., Sultan, M., Thomas, M. L., Vaghar-Kashani, A. & Marcato, P. Retinoid signaling in cancer and its promise for therapy. *J. Carcinog. Mutagen*. S7 (2013).

[33] Centritto F (2015). Cellular and molecular determinants of all-trans retinoic acid sensitivity in breast cancer: Luminal phenotype and RARα expression. EMBO Mol. Med..

[34] Liu R-Z (2016). Association between cytoplasmic CRABP2, altered retinoic acid signaling, and poor prognosis in glioblastoma. Glia.

[35] Liu R-Z (2015). CRABP1 is associated with a poor prognosis in breast cancer: adding to the complexity of breast cancer cell response to retinoic acid. Mol. Cancer.

[36] Engedal N, Auberger P, Blomhoff HK (2009). Retinoic acid regulates Fas-induced apoptosis in Jurkat T cells: reversal of mitogen-mediated repression of Fas DISC assembly. J Leukoc Biol.

[37] Toth R (2001). Activation-induced apoptosis and cell surface expression of Fas (CD95) ligand are reciprocally regulated by retinoic acid receptor α and γ and involve nur77 in T cells. Eur, J. Immunol..

[38] Bissonnette RP (1995). 9-cis retinoic acid inhibition of activation-induced apoptosis is mediated via regulation of fas ligand and requires retinoic acid receptor and retinoid X receptor activation. Mol. Cell. Biol..

[39] Xia S-L (2015). CRABP-II and FABP5 independent responsiveness of human glioblastoma cells to all-trans retinoic acid. Oncotarget.

[40] Donato LJ, Noy N (2005). Suppression of Mammary Carcinoma Growth by Retinoic Acid: Proapoptotic Genes Are Targets for Retinoic Acid Receptor and Cellular Retinoic Acid–Binding Protein II Signaling. Cancer Res..

[41] Dhanasekaran DN, Reddy EP (2008). JNK signaling in apoptosis. Oncogene.

[42] Ichijo H (1997). Induction of apoptosis by ASK1, a mammalian MAPKKK that activates SAPK/JNK and p38 signaling pathways. Science.

[43] Nishitoh H (1998). ASK1 is essential for JNK/SAPK activation by TRAF2. Mol. Cell.

[44] García S, Liz M, Gómez-Reino JJ, Conde C (2010). Akt activity protects rheumatoid synovial fibroblasts from Fas-induced apoptosis by inhibition of Bid cleavage. Arthritis Res Ther.

[45] Itoh K, Hase H, Kojima H, Saotome K, Nishioka K (2004). Central role of mitocondria and p53 in Fas-mediated apoptosis of rheumatoid sinovial fibroblasts. Rheumatology.

[46] Tran SE, Holmström TH, Ahonen M, Kähäri V-M, Eriksson JE (2001). MAPK/ERK overrides the apoptotic signaling from Fas, TNF, and TRAIL receptors. J. Biol. Chem..

[47] Holmström TH (2000). MAPK/ERK signaling in activated T cells inhibits CD95/Fas-mediated apoptosis downstream of DISC assembly. EMBO J..

[48] Orosa B, Gonzalez A, Mera A, Gómez-Reino JJ, Conde C (2012). Lysophosphatidic acid receptor 1 suppression sensitizes rheumatoid fibroblast-like synoviocytes to TNF-induced apoptosis. Arthritis Rheum..

[49] Bartok B, Firestein GS (2010). Fibroblast-like synoviocytes: key efector cells in rheumatoid arthritis. Immunol. Rev..

[50] Garcia S, Bodaño A, Pablos JL, Gomez-Reino JJ, Conde C (2008). Poly(ADP-ribose) polymerase inhibition reduces tumor necrosis factor-induced inflammatory response in rheumatoid sinovial fibroblasts. Ann. Rheum. Dis..

[51] Hong S (2015). TNF-α confers resistance to Fas-mediated apoptosis in rheumatoid arthritis through the induction of soluble Fas. Life Sci..

[52] Kim W-U (2006). Hydroxychloroquine potentiates Fas-mediated apoptosis of rheumatoid synoviocytes. Clin. Exp. Immunol..

[53] Peng SL (2006). Fas(CD95)-related apoptosis and rheumatoid arthritis. Rheumatology.

[54] Arnett FC (1987). The American Rheumatism association1987 revised criteria for the classification of rheumatoid arthritis. Arthritis Rheum.

[55] Vreeland AC, Levi L, Zhang W, Berry DC, Noy N (2014). Cellular retinoic acid-binding protein 2 inhibits tumor growth by two distinct mechanisms. J. Biol. Chem..

[56] Fischer-Huchzermeyer S (2017). The celular retinoic acid binding protein 2 promotes survival of maligant peripheral nerve sheat tumor cells. Am. J. Pathol..

