# Supplementary material for: Uncovering Cellular retinoic acid-binding protein 2 as a potential target for rheumatoid arthritis synovial hyperplasia
Source: Sci Rep. 2018 Jun 7;8:8731. doi: 10.1038/s41598-018-26027-x (PMC5992205; doi:10.1038/s41598-018-26027-x)
Supplement: Supplementary file 1 — Dataset 1 [file 41598_2018_26027_MOESM1_ESM.docx]

**Supplementary Information**

**Uncovering Cellular retinoic acid-binding protein 2 as a potential target for rheumatoid arthritis synovial hyperplasia**

Nerea Mosquera, Angela Rodriguez-Trillo, Antonio Mera-Varela, Antonio Gonzalez, Carmen Conde.

Laboratorio de Reumatología Experimental y Observacional, y Servicio de Reumatología, Instituto de Investigacion Sanitaria de Santiago (IDIS), Hospital Clínico Universitario de Santiago de Compostela (CHUS), SERGAS. Travesía da Choupana s/n, Santiago de Compostela, 15706, Spain.


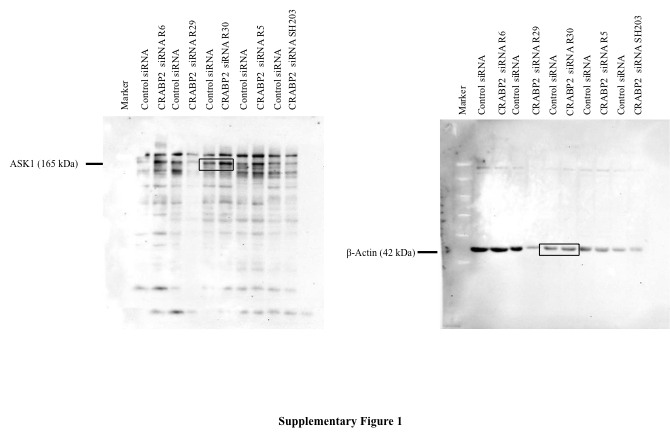


**Supplementary Figure 1.** Uncropped western blot of Figure 4B.


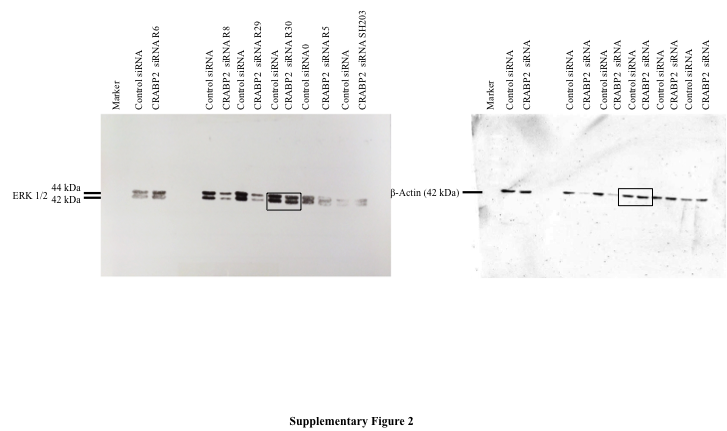


**Supplementary Figure 2.** Uncropped western blot of Figure 4C.


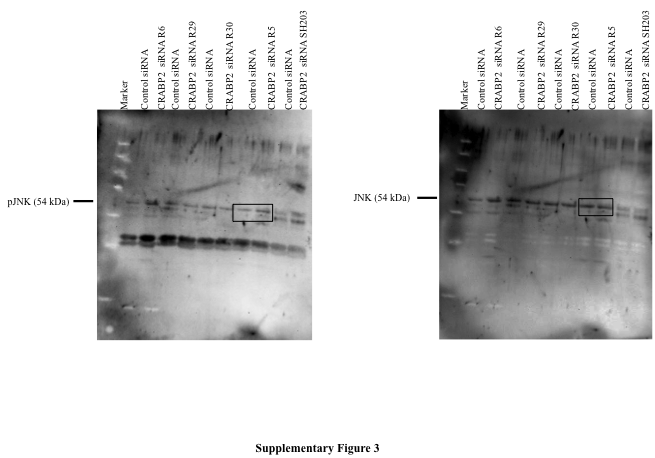


**Supplementary Figure 3.** Uncropped western blot of Figure 4D.
